# The TALENT II study: a randomized controlled trial assessing the impact of an individual health management (IHM) on stress reduction

**DOI:** 10.1186/s12889-018-5756-3

**Published:** 2018-07-04

**Authors:** Dieter Melchart, Erich Wühr, Kristina Wifling, Beatrice E. Bachmeier

**Affiliations:** 1 Director of the Competence Center for Complementary Medicine and Naturopathy (CoCoNat), Klinikum rechts der Isar, Technische Universität München, D-80801 Munich, Germany; 2 Institute for Complementary and Integrative Medicine, University Hospital Zurich and University of Zurich, CH-8091 Zurich, Switzerland; 3 Member of the faculty for Applied Health Care Science, Deggendorf Institute of Technology DIT, D-94469 Deggendorf, Germany; 4 Competence Center for Complementary Medicine and Naturopathy (CoCoNat), Klinikum rechts der Isar, Technische Universität München, D-80801 Munich, Germany; 5 Institute of Laboratory Medicine, University Hospital, LMU Munich, Munich, Germany

**Keywords:** Stress, Burnout, Prevention, Intensive lifestyle intervention, Individual health management (IHM), E-health, Yang sheng, Traditional Chinese medicine (TCM)

## Abstract

**Background:**

Unfavorable lifestyle factors influence the risk of stress disorders. For risk reduction, lifestyle modifications, such as regular physical activity, balanced nutrition and competence in stress management, are a means of choice. The clinical study examines the efficacy of an intensive lifestyle intervention, named Individual Health Management (IHM), − with regard to a reduction of perceived stress. The study is supported by the major regional health insurance, which conducts, in cooperation with the Traditional Chinese Medicine (TCM) hospital, Bad Kötzting, a local model project offering insurants the IHM program as prevention measure against stress and related aftermath.

**Methods:**

The study is a controlled, randomized, monocentric trial with a 12-months intervention phase. Feasible persons are checked according to inclusion and exclusion criteria and assigned to the intervention or control group. Randomization ratio is 1:1. (A) Participants of the intervention group receive the lifestyle program IHM, have access to a web-based health portal (www.viterio.de), and join 3 full-day and 10 two-hour training sessions during the first 3 months. During the remaining 9 months, 4 training sessions and a weekly monitoring are performed with remote assistance. Besides measurement of perceived stress, examinations include burnout symptoms, heart rate variability, laboratory and physical findings. Further patient reported outcomes are documented (e.g. well-being, life satisfaction, severity of mood state, sense of coherence, psycho-vegetative test, cardio-metabolic risk factors, hypertension and diabetes risk. (B) Participants in the control group have access to the intensive lifestyle intervention IHM after a waiting period of at least 6 months. Examinations are conducted at baseline, after 3 and 6 months and in the intervention group additionally after 9 and 12 months. The confirmatory analysis examines the differences between the two groups with regard to changes in perceived stress after 6 months compared to the initial value.

**Discussion:**

In order to enhance adherence, avoid attrition and to insure data quality, different measures will be implemented in the study. Based on a blended learning concept including a web-based e-health tool named VITERIO®, the program promises to achieve sustainable effects in perceived stress.

**Trial registration:**

German Clinical Trial Register Freiburg (DRKS): DRKS00013040 (date registered 2017–10-1).

**Electronic supplementary material:**

The online version of this article (10.1186/s12889-018-5756-3) contains supplementary material, which is available to authorized users.

## Background

The subjective perception of excessive demands is discussed as causing a variety of diseases [1]. In this context chronic stress can lead to the development of a burnout syndrome [2] as well as overweight [3], high blood pressure [4] and diabetes [5]. For the indication “overweight”, the effects of lifestyle intervention on sustainable weight reduction (1 year and longer) are well documented in literature, like e.g. the “Look AHEAD” (action for health in diabetes) project [6] or “Guideline for the management of overweight and obesity in adults” [7]. Using the keyword “stress reduction” a literature search reveals predominantly studies, which concentrate their programs and techniques merely on the reduction of stress per se, rather than to change the whole life as practiced in intensive lifestyle intervention programs. In contrast a multimodal stress prevention program as executed in a very recently published randomized controlled study by Stier-Jarmer and coworkers, showed remarkably positive effects after 6 months in the intervention group as opposed to the control group [8]. A systematic review of 25 studies showed that 80% of all interventions had positive effects on burnout. The supply of refresher seminars intensified the success of the programs [9].

Main objective and target parameter of the study will be stress-reduction, although the basic concept of IHM (Individual Health Management) is very broad and multifaceted [10]. The sustainability of IHM has already been proven due to an all-embracing change in lifestyle [11, 12].

The stress prevention study is embedded into a comprehensive network program whose objective is to facilitate and to conduct prevention programs for different indications and risk groups at selected spa regions in Bavaria. Thereby the medical quality of health services offered in the spas shall be improved.

The study is supported by the major regional health insurance, AOK Bayern (Allgemeine Ortskrankenkasse - General Health Insurance, Bavaria), which conducts, in cooperation with the TCM hospital, Bad Kötzting, a local model project offering insurants the IHM program as preventive measure against stress and related aftermath. The outcomes are expected to have high impact on health policy, as the study is designed to provide evidence that perceived stress can be remarkably reduced already after 1 year of IHM lifestyle intervention. In case of positive outcomes a preventive effect on stress related diseases can be suggested, which will ultimately relieve health insurance providers.

In this context a randomized controlled trial on weight reduction (TALENT study) has been performed previously by our group [12, 13] resulting in a statistically significant weight reduction of about 10% of the baseline weight after one year [12]. Our own preliminary data showed our concept for a web-based lifestyle intervention program as applied throughout the IHM is a can be successfully implemented in a specialized spa region with suspected effectiveness in people with perceived stress [14]. The program has been conducted in the Bavarian health resort area of Bad Kötzting, which has become a center of preventive medicine – also due to the reason that the first German hospital for Traditional Chinese Medicine (TCM) is located there.

The comprehensive lifestyle intervention program IHM which has initially been established as a framework program for mainstream and complementary approaches of lifestyle medicine was developed at the Competence Center for Complementary Medicine and Naturopathy (CoCoNat) of the TU Munich, Germany. Basic medical experience, technical prerequisites and training resources necessary for the accomplishment of the IHM are on location. Development of the intensive lifestyle intervention was supported by public funding provided by the Bavarian Federal Health Office. By making use of both conventional and TCM methods, our comprehensive lifestyle intervention program addresses primarily all persons who would like to activate the body’s own resources and health supporting potential for self-healing [15].

The IHM should enable the participant to learn how to manage own stress situations through regulation of emotions. This should result in a reduction of the perceived stress levels.

Aim of the here presented study is to evaluate the efficacy of the lifestyle intervention program IHM in reduction of perceived stress in respect to a control group (waiting list) after 6 months. Additionally, within the IHM intervention group, the further course until the end of the lifestyle intervention program after 12 months will be analyzed.

## Methods

### Study design

The study is a monocentric randomized controlled trial. The study duration for each participant is 6 months. In the intervention group there is also a longitudinal analysis with examinations after months 9 and 12. The study will be performed at the SINOCUR prevention center in Bad Kötzting, Germany. The center is committed to be part of the core teams promoting Individual Health Management (IHM) which are pooled into a centrally coordinated network of health promotion called “IHM campus”.

### Web-based recruitment and participants

For recruitment of participants The AOK Bayern Health insurance contacted < 60.000 authorized insurants in the catchment area of the IHM Prevention center SINOCUR in Bad Kötzting, via written correspondence. The contacted persons are invited to conduct a short health survey in the e-Health portal VITERIO® which is part of the IHM [10]. At the end of the survey all interested persons can decide whether to take part in the study or not. The persons who are willing to participate in the study will contact the local IHM team in Bad Kötzting which will provide basic information around the study and perform the screening for the main inclusion criteria. A personal appointment with all persons who apparently will comply with the requirements of the study is planned and the trial physician will check all criteria for inclusion and exclusion after a comprehensive examination of all potential participants. Information on background, objectives benefits and risks of the study will be provided by the trial physician by a leaflet as well as orally.

### Inclusion/exclusion criteria

Persons of both sexes, aged 18–70 years resident in the catchment area of Bad Kötzting, with Tedium-Measure ≥3,20 (= moderate stress, pre-burnout), Perceived Stress Questionnaire (PSQ) total score > 41, and subjective feeling of stress exposure for more than 3 months, can be included in the study. A written informed consent is mandatory.

Persons will not be included if one of the following exclusion criteria is present: being unable to consent for themselves, insufficient skills in German language, no private access to internet, known hypertension (systolic blood pressure ≥ 160 mmHg or diastolic blood pressure ≥ 100 mmHg) with or without medication, known hypotension (systolic blood pressure ≤ 70 mmHg or diastolic blood pressure ≤ 50 mmHg), body mass index (BMI) < 17,5, psychiatric/psychotherapeutic treatment requirements defined as “severe signs and symptoms” according to the ISR (ICD-10-Symptom Rating) questionnaire in at least one of the subscales depressive syndrome, anxiety syndrome, obsessive-compulsive syndrome, somatoform syndrome, eating disorder syndrome or in the ISR total score, known psychiatric treatment (with or without medication), known diabetes mellitus (type 1 or 2), known heart disease (like CHD, arrhythmia, valvular heart defect, cardiac insufficiency), known gastric or duodenal ulcers, known diseases of the liver or kidneys, known diseases of the eyes (e.g. retinal detachment), known disease of the thyroid gland or taking of thyroid hormones, known disease-related impairments preventing a participation in the lifestyle program (e.g. arthrosis), known therapeutic conditions that are due to known risks/side effects not compatible with participation in the lifestyle program, known pregnancy (or lactation) or planned in the next year, participation in another currently clinical trial or during the last 6 months.

### Number of cases

Command variable was the change in overall perceived stress as evidenced by means of PSQ total score in month 6 in comparison to base line at month 0 (M0) collected as continuous numerical value.

For PSQ base line values over 41 mean reduction of overall PSQ levels after 6 months is expected to be 18 points in the intervention group and 9 points in the control group according to our own pilot study [14]. This corresponds to a 33% reduction of base line levels in the intervention group. Mean variation in PSQ value change was assumed to be 18 points in both groups. Therefore, the sample size for the t-test for independent groups (□ = 0.05, two-sided, power 80%, □ = 0.2) was estimated as 64 in each group (allocation ratio 1:1). Taking into account an expected dropout rate of 5%, a total sample size of 136 participants was deemed appropriate.

### Randomization

Randomization will be carried out as previously described by us [13]. Briefly, participants will be randomized after formal inclusion. Closed envelopes will be opened by the trial physician in strictly sequential order of the enrolments and the allocated study arm will be disclosed to the study participant. The allocation ratio is 1:1, means 1 for the intervention group and 1 for the control group (waiting list). Randomization will be prepared for 150 participants (including replacement numbers). An independent data manager at the institute for medical statistics and epidemiology at the TU Munich will prepare randomization and allocation envelopes.

### Intervention

The study compares an intervention (IHM = Individual Health Management) with a control group (6 months waiting list for IHM).

**Fig. 1:**
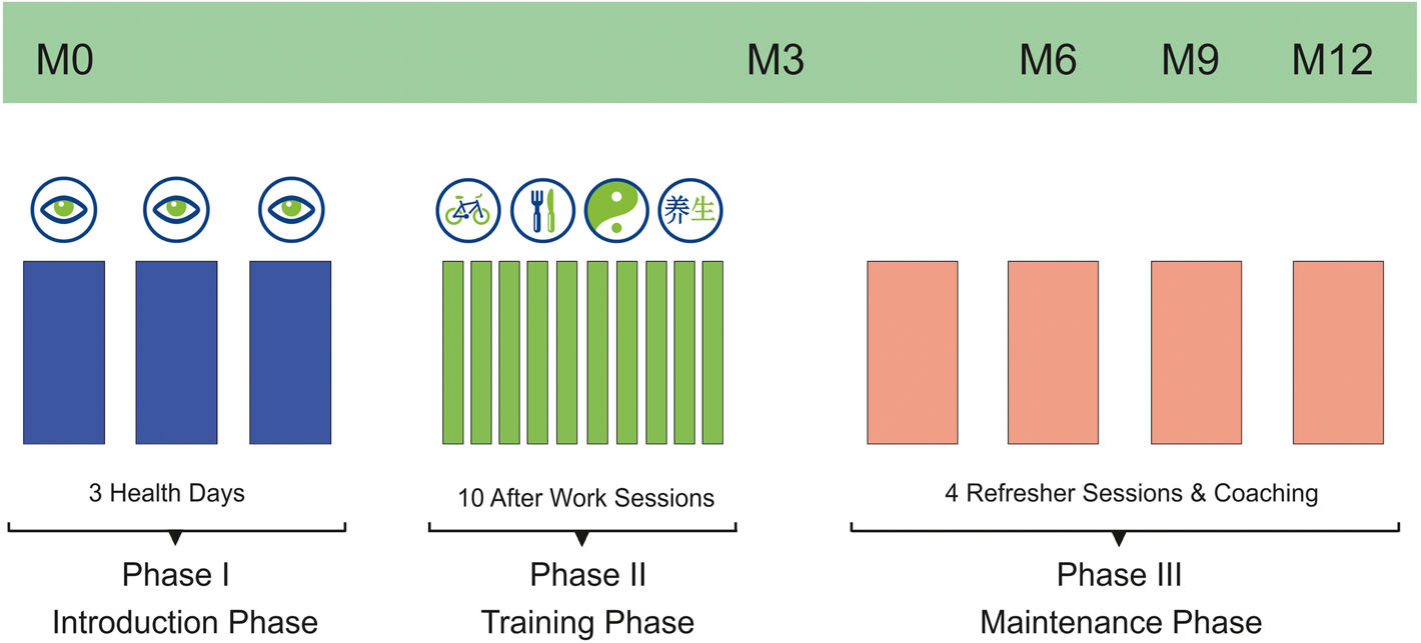
Course of action intervention group

Group IHM: The lifestyle intervention program IHM is a therapeutic intervention against stress, pre-burnout and burnout. It consists of several phases and training packages and spans over a time period of 12 months, including intensive lifestyle counseling during the first three months. The basic training consists of an introduction phase with 3 health days and a training phase with ten weekly after-work group seminars. Subsequently the intervention program envisages a 9-months maintenance phase with weekly monitoring by specially trained health coaches and as needed distant lifestyle counseling, as well as trimestral refresher days (Fig. 1).

In particular the introduction phase consists of three full health days in which the participants learn how to practice self-awareness (individual life conditions and their impact on the health status), how to optimize quality of life and life satisfaction as well as the proper TCM constitution type. The participants are trained how to use the e-Health portal VITERIO® and how to perform 6-min- and 2-km-walking-tests and basic knowledge on “3–1-2” QiGong relaxation technique among others. Further training contents are techniques of time management, information on stress and resilience, regulation of mood and exertion, the significance of dietary days for stress reduction, knowledge of the individual risk and protection factors, and to determine individual aims in life.

During the intervals of the three health days, participants are encouraged to perform self-observation and to document their lifestyle in the VITERIO® e-Health portal, which performs an evaluation in form of a so-called time-and-mood analysis. The results shall enhance the participant’s sense of self. Additionally health behavior in regard to movement, nutrition and stress will be promoted. By means of systematic feedback to VITERIO® the participants’ changes in lifestyle, behavior and regulation of emotions will be recorded and evaluated.

The 3-month-training-phase encompasses 10, not less than 2-h after work training sessions in which the participant practices health behavior, in particular mental resilience and handling of stress, relaxation techniques, and how to plan “being and staying healthy” with medical check-ups and priority lists. There will be lectures on time management, nutrition and deeper insights into QiGong.

During the subsequent 9-month-maintenance phase, the participants are monitored on a weekly base by the health coaches. Via a PROFI (provider reported outcomes of findings and interventions) access to VITERIO® the Coach has insight into the individual health data entered by the participant into the e-health portal. Thereby changes in perceived stress or weight can be detected and analyzed in depth. On demand this type of distant life style counseling can be supplemented by personal training sessions. 4 refresher sessions within 9 months shall be performed in order to deepen and substantiate teaching and learning contents.

Based on the documented health data, the health coach provides a final assessment that reveals the achievement of the participant’s goals at the end of the intervention.

**Fig. 2:**
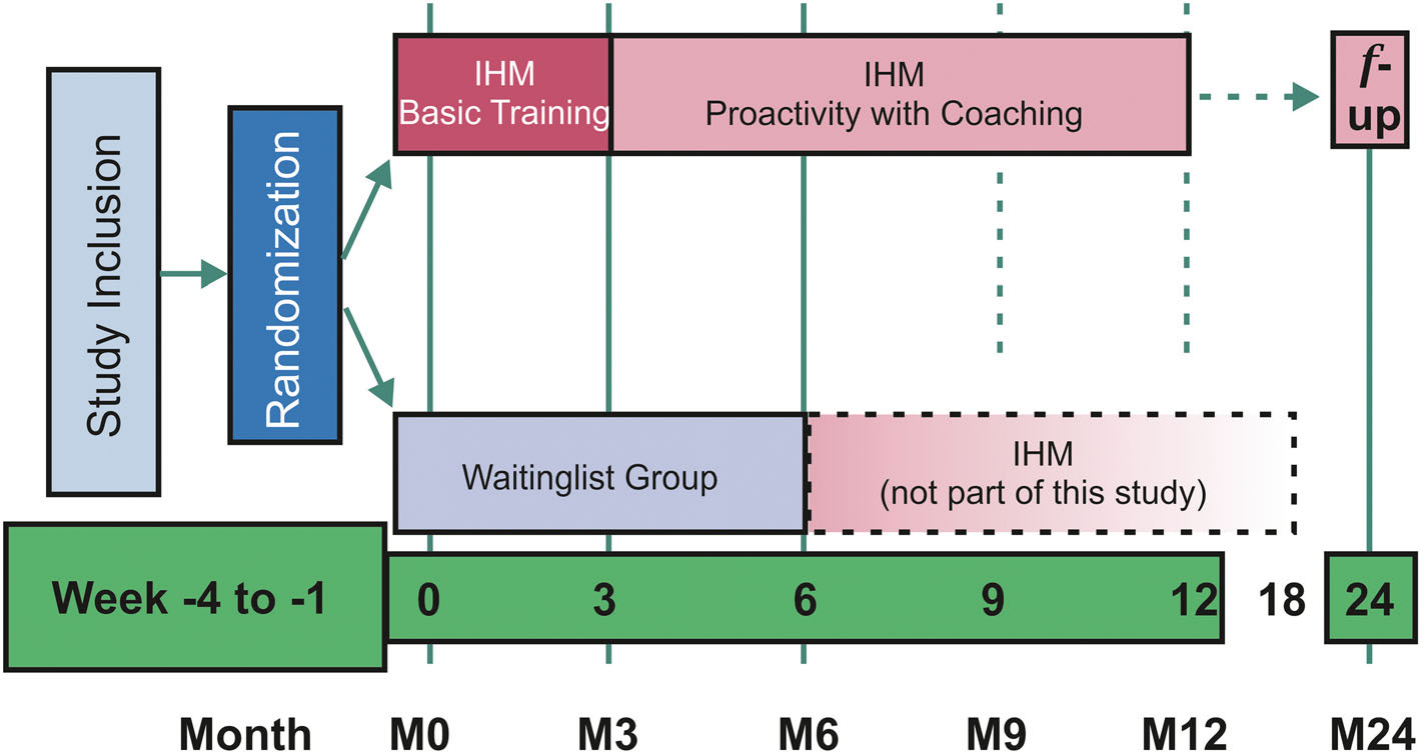
Flow chart showing the design of the study (IHM = Individual Health Management; SEPP = Sino-European-Prevention-Program)

Figure 2 shows the basic design of the study with the two study groups. Table 1 gives an overview on the study process.

**Table 1:** Study Process Chart

	Month				
Examination	0	3	6	9	12
Inclusion−/exclusion criteria	X				
Informed consent	X				
Randomization	X				
Sociodemographic data	X				
Size	X				
Weight, waist circumference, Body-Mass-Index, blood pressure, heart rate	X	X	X	X	X
Laboratory findings:					
- Fasten glucose	X	X	X	X	X
- Triglycerides	X	X	X	X	X
- Total cholesterol	X	X	X	X	X
- HDL−/LDL-Cholesterol	X	X	X	X	X
- Kidney (Crea, Urea)	X		X		X
- Liver (ASAT, ALAT, GGT, ALP, Bilirubin)	X		X		X
- Electrolytes (Ca, K, Cl, Mg, Na)	X		X		X
- Blood count (Ery, Leuco, Ptl, Hk, Hb)	X		X		X
- TSH (basal)	X		X		X
Perceived Stress Questionnaire (PSQ)	X	X	X	X	X
ICD-10-Symptom Rating (ISR)	X	X	X	X	X
Heart Rate Variability (HRV)	X	X	X	X	X
TCM-Diagnosis	X	X	X	X	X
Adverse events/effects		X	X	X	X

### Outcomes

The primary outcome measure to test the hypothesis of no differences between the effects of both groups in perceived stress (PSQ total score; Perceived Stress Questionnaire) from baseline to month 6. Secondary outcome parameters are Tedium-Measure, ICD-10-Symptom-Rating (ISR), TCM diagnosis, TCM constitution, heart rate variability (HRV), heart rate, laboratory findings, waist circumference, blood pressure, stresses and strains, 3-level-stresstest, severity of mood state in general (VAS), life satisfaction (FLZ), well-being and vitality (WHO-5-well-being), self-efficacy, optimism and pessimism (SWOP), motivation and willingness to change, sense of coherence (SOC-13), social support (SSS), psycho-vegetative test, comprehensive medical history, cardiovascular and metabolic risk factors, hypertension risk, diabetes risk (FINDRISK), nutrition index, moving index, physical power, and the body’s defenses. The occurrence of adverse events will be captured systematically at each physical examination following the baseline testing. A list of all questionnaires and the time points of data collection are given in Table 2.

**Table 2:** Questionnaires

	Month				
Health Check	0	3	6	9	12
Health Check 1					
- Comprehensive medical history	X	X	X	X	X
- Hypertension risk	X	X	X	X	X
- Diabetes risk (FINDRISK)	X	X	X	X	X
- Motivation and willingness to change	X		X		X
- Life satisfaction (FLZ)	X	X	X	X	X
- Burnout Scale (Tedium-Measure)	X	X	X	X	X
- TCM constitution	X	X	X	X	X
Health Check 2					
- Psycho-vegetative test	X	X	X	X	X
- Severity of mood state in general (VAS)	X	X	X	X	X
- Stresses and strains	X	X	X	X	X
- Well-being and vitality (WHO-5-well-being)	X	X	X	X	X
- Cardiovascular and metabolic risk factors	X	X	X	X	X
- Self-efficacy, optimism and pessimism (SWOP)	X	X	X	X	X
- 3-level-stresstest	X	X	X	X	X
- Nutrition index	X	X	X	X	X
- Moving index	X	X	X	X	X
- Physical power	X	X	X	X	X
- The body’s defenses	X	X	X	X	X
- Sense of coherence (SOC-13)	X	X	X	X	X
- Social support (SSS)	X	X	X	X	X

### Statistical analysis

The confirmatory analysis will be conducted using a 2-sided significance test at the 5% significance level. Based on the intent-to-treat population (ITT) the primary outcome (change in perceived stress (PSQ total score) Δmonth 0 – month 6) will be tested by analysis of variance with the grouping factor “intervention” controlled for baseline value. In participants with missing data for PSQ total score at month 6 (drop-outs) an adequate conservative imputation technique will be applied. Secondary endpoints will be analyzed analogously but using an explorative approach.

All captured data will be analyzed descriptively by appropriate statistical parameters: absolute and relative frequencies for categorical data and arithmetic means, medians, standard deviations for numerical data. Where indicated, 95% confidence intervals will be presented. Apart from the confirmatory analysis a series of sensitivity analyses will be performed to explore the impact of different factors on the results of the analyses. Relevant factors are among others adherence to the protocol, adherence to the intervention program IHM and different responder definitions.

In terms of sensitivity analysis, primary and secondary endpoints will be examined based on the ITT as well as the per-protocol (PP) population. The ITT population will comprise all patients that had compiled the PSQ total score at least at baseline. They will be evaluated as randomized. Subjects will be considered PP if they had participated in the study according to the protocol.

### Ethical review

Ethical approval for this study was obtained from the ethics commission of the Medical Faculty of TU Munich (file number 278/17S). The investigators will ensure that the study will be conducted in compliance with the ethical guidelines as set out by this committee, and in line with the guidelines for good clinical practice (GCP).

## Discussion

The comprehensive lifestyle intervention program IHM, was designed to enable people to manage sufficiently their own stress situations. In this context the capacity to regulate emotions is an important technique to reduce perceived stress levels. Differently from other lifestyle interventions like e.g. in weight reduction programs, where objective measurands like weight or BMI indicate the success of the program, stress levels and their impact on health state can only be evaluated as experienced by the individual. To measure stress levels. we use the perceived stress questionnaire (PSQ) developed by Levenstein et al. in 1993 [16] and modified by Fliege et al. in 2005 [17]. The PSQ with its high consistency, high reliability and validity is proven to be superior to alternative measures for predicting stress-related health outcomes. In a previously conducted pilot-study, we considered exhaustion as an important symptom of stress and measured sub dimensions like fatigue/loss of motivation (Tedium Measure), low vitality (WHO-5-well-being index) and sleep disturbance (neuro-vegetative questionnaire) as indicators for the degree of exhaustion. After 6 months of comprehensive lifestyle intervention by IHM all indicators for stress-based disorders showed relevant improvements in a consistent way [14].

The implementation of IHM helps participants to strengthen self-perception and self-reflection and gives support to avoid inadequate emotional-cognitive assessments and maladaptive behavior in order to stay healthy. This is achieved by organismic and psycho-social, as well as cognitive contributions, which are a core issue of complementary medicine. The strengthening of salutogenic resources and protective factors for health maintenance are pro-active processes that needs a strong active involvement of the individual. Implementation of comprehensive lifestyle intervention concepts in routine care requires a difficult interplay of statutory sickness funds, physicians, coaches and the participants themselves. The performance of a randomized controlled trial (RCT), as presented here, is of utmost importance to provide convincing data to all partners involved.

### Quality assurance and bias

The protocol was developed according to the Consort guidelines (see Additional files 1 and 2). Qualification of the trial physicians regarding the legal requirements was according to the German Drug Law (AMG) and in line with the guidelines for GCP. Personnel in the study center and the participating spa regions being involved in coaching of IHM participants have been trained and certified as designated IHM health coaches. Monitoring of the study comprising central monitoring as well as onsite visits at the participating study centers will be executed by a professional institution with acknowledged expertise in supervision of clinical trials (Munich Study Center at the Medical faculty of TUM). An independent statistician at the institute for medical statistics and epidemiology at the TUM will conceptualize and perform the confirmatory analysis of the data. In order to avoid early drop-outs comprehensive information on the study will be handed out to each participant.

Participants allocated to the IHM study arm will follow a lifestyle intervention program aimed to reduce stress, while participants in the control group are on a waiting list for 6 months before they will also enter the IHM intervention. The comprehensive lifestyle intervention IHM is requesting a high level of active participation and the potential effectiveness of an intervention is affected by non-adherence to the program. All potential study participants are informed in detail on the study requirements before enrollment. During the 6 months-study duration there are sixteen personal meetings scheduled and it has to be expected that especially for those participants with a longer distance between their domicile and the study center not all appointments will be followed. We will implement a simple score for compliance based on the rate of participation and will use this for sensitivity analyses to check for potential association with effect sizes. All steps and phases of IHM are clearly predefined and therefore the individual health behavior actually practiced in the participants’ everyday life has to be analyzed and adapted to the program. Concerning participants in the intervention group of the trial: Changes in life situations, personal crisis as well as success can be the cause of alterations in lifestyle, however this will not automatically lead to an exclusion from the program or the trial. Similarly, participants of the control group who apparently improve their lifestyle only by written advice need not be excluded.

### Expected benefit

This randomized controlled study will contribute to the evidence of a comprehensive lifestyle program which in the current form has not yet been proved for stress reduction. The program with an active duration of 12 months promises to achieve sustainable effects in this context. Based on a blended learning concept and using web-based e-health tools the program might overcome the well-known challenges of many stress-relief programs. Although knowing how lifestyle and self-awareness should be modified it proves to be hard to sustainably improve health related behavior in everyday life. Lifestyle intervention programs including refresher seminars are expensive due to personal lifestyle counseling by specially trained coaches, and costly data management. Many persons suffering from stress or overweight cannot afford to participate in these programs and so far health insurances do not reimburse the charges. The major regional health insurance, AOK Bayern, is the first to support a lifestyle intervention program in order to make it available as model project for their insurants. It is conceivable that, in future, this preventive measure against stress and obesity related diseases will be an inherent part of reimbursement policy of health insurances in case this model project proofs to be successful.

Furthermore, our study will show whether comprehensive lifestyle training can be implemented successfully in local health and prophylaxis centers. The here presented clinical trial is designed monocentric, however the idea can be easily transferred to various other health centers and spas. A pertinent concept to transfer the idea of IHM to other spa regions is already existing in the IHM campus, which is a comprehensive network to install and maintain prevention programs for different risk groups at selected spas in Bavaria. This could be the door opener to make a nationwide shift in paradigm from a disease-orientated medical care to a salutogenic based health policy supporting prevention and maintaining public health.

## Additional files

Additional file 1: CONSORT 2010 Checklist (DOC 215 kb)

Additional file 2: CONSORT 2010 Flow Diagram (DOC 48 kb)

## Abbreviations

AHEAD: Action for Health in Diabetes
AMG: Arzneimittelgesetz (German Drug Law)
AOK Bayern: Allgemeine Ortskrankenkasse - General Health Insurance, Bavaria
BMI: Body Mass Index
CHD: Coronary Heart Disease
CoCoNat: The Competence Center for Complementary Medicine and Naturopathy
FINDRISK: Finnish Diabetes Risk Study
FLZ: Fragebogen zur Lebenszufriedenheit (questionnaire on life satisfaction)
GCP: Good Clinical Practice
IHM: Individual Health Management
ISR: ICD-10-Symptom Rating
ITT: Intend-to-Treat
PP: Per Protocol
PROFI: Provider Reported Outcomes of Findings and Interventions
PSQ: Perceived Stress Questionnaire
RCT: Randomized Controlled Trial
SOC: Sense of Coherence
SSS: Social Support Scale
SWOP: Self-efficacy, Optimism and Pessimism
TALENT: TAilored Lifestyle IntervENTion
TCM: Traditional Chinese Medicine
TU: Technical University
VAS: Visual Analogue Scale
VITERIO: A VIrtual Tool for Education, Reporting, Information and Outcomes
