# Supplementary material for: The TALENT II study: a randomized controlled trial assessing the impact of an individual health management (IHM) on stress reduction
Source: BMC Public Health. 2018 Jul 4;18:823. doi: 10.1186/s12889-018-5756-3 (PMC6030744; doi:10.1186/s12889-018-5756-3)
Supplement: Supplementary file 2 — CONSORT 2010 Flow Diagram (DOC 48 kb) [file 12889_2018_5756_MOESM2_ESM.doc]

**
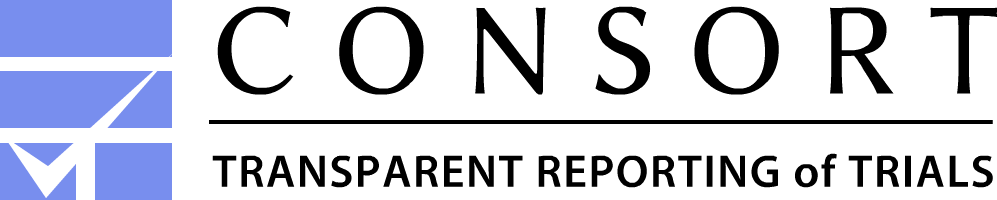
**

**CONSORT 2010 Flow Diagram**

**Allocation**

**Analysis**

**Follow-Up**

**Enrollment**

Assessed for eligibility (n= )

Excluded (n= )

♦  Not meeting inclusion criteria (n= )

♦  Declined to participate (n= )

♦  Other reasons (n= )

Analysed (n= )
♦ Excluded from analysis (give reasons) (n= )

Lost to follow-up (give reasons) (n= )

Discontinued intervention (give reasons) (n= )

Allocated to intervention (n= )

♦ Received allocated intervention (n= )

♦ Did not receive allocated intervention (give reasons) (n= )

Lost to follow-up (give reasons) (n= )

Discontinued intervention (give reasons) (n= )

Allocated to intervention (n= )

♦ Received allocated intervention (n= )

♦ Did not receive allocated intervention (give reasons) (n= )

Analysed (n= )
♦ Excluded from analysis (give reasons) (n= )

Randomized (n= )
